# Functional maturation of cytochromes P450 3A4 and 2D6 relies on GAPDH- and Hsp90-Dependent heme allocation

**DOI:** 10.1016/j.jbc.2024.105633

**Published:** 2024-01-08

**Authors:** Sidra Islam, Dhanya Thamaraparambil Jayaram, Pranjal Biswas, Dennis J. Stuehr

**Affiliations:** Department of Inflammation and Immunity, Lerner Research Institute, The Cleveland Clinic, Cleveland, Ohio, USA

**Keywords:** heme protein, chaperone, iron, heme trafficking, monooxygenase, drug metabolism

## Abstract

Cytochrome P450 3A4 and 2D6 (EC 1.14.13.97 and 1.14.14.1; CYP3A4 and 2D6) are heme-containing enzymes that catalyze the oxidation of a wide number of xenobiotic and drug substrates and thus broadly impact human biology and pharmacologic therapies. Although their activities are directly proportional to their heme contents, little is known about the cellular heme delivery and insertion processes that enable their maturation to functional form. We investigated the potential involvement of GAPDH and chaperone Hsp90, based on our previous studies linking these proteins to intracellular heme allocation. We studied heme delivery and insertion into CYP3A4 and 2D6 after they were transiently expressed in HEK293T and GlyA CHO cells or when naturally expressed in HEPG2 cells in response to rifampicin, and also investigated their associations with GAPDH and Hsp90 in cells. The results indicate that GAPDH and its heme binding function is involved in delivery of mitochondria-generated heme to apo-CYP3A4 and 2D6, and that cell chaperone Hsp90 is additionally involved in driving their heme insertions. Uncovering how cells allocate heme to CYP3A4 and 2D6 provides new insight on their maturation processes and how this may help to regulate their functions in health and disease.

Cytochrome P450’s (CYP’s) are a large family of iron-protoporphyrin IX (heme)-containing enzymes that catalyze the oxidation of a wide variety of natural compounds and xenobiotics (1). Their activities directly depend on their heme content due to its binding and activating dioxygen to ultimately form the reactive heme-peroxo and -ferryl species involved in substrate oxidations (2). In mammals, certain CYP’s are expressed in relative abundance in the liver where they are utilized to oxidize xenobiotics and drugs to aid in their elimination (3, 4), while other CYP’s are expressed throughout the body to help generate arachidonate metabolites that have broad biological impacts (5, 6). Of the former group, CYP3A4 and 2D6 are hallmark members that are primarily expressed in the liver and have been extensively studied. Their expression can be further induced by exposure to steroids and different foreign compounds, and like most CYP’s, they primarily localize to the endoplasmic reticulum (ER) *via* an N-terminal anchoring sequence, leaving a portion of the CYP protein exposed to the cytosol (7, 8, 9, 10, 11). CYP3A4 and 2D6 are of primary importance to pharmacology because their promiscuous substrate binding properties and relatively high expression levels in the liver allows their activities to determine the metabolism and efficacy of many pharmaceuticals (12).

In contrast to the extensive research on CYP expression, catalysis, and pharmacology, relatively little is known about how CYP enzymes mature to their functional forms in cells. In particular, it is still unclear how heme generated in the mitochondria is delivered and inserted into CYP proteins. Early studies suggested that CYP heme acquisition might occur in concert with their protein expression (13), but other studies indicated that CYP proteins may build up in their heme-free forms during their maturation (14, 15), particularly in tissues other than the liver including the brain, lung, and kidney (16, 17). Given the propensity of CYP’s to attach to the ER, it has also been suggested that heme delivery to such proteins might occur through mitochondrial-ER membrane contact (18). We thus sought to identify cellular mechanisms that provide heme to human CYP3A4 and 2D6.

Our recent studies on heme protein maturation have shown that GAPDH, *via* its specific heme binding ability, is required for mitochondrial heme allocation to several heme proteins including NO synthases, hemoglobin (Hb), myoglobin (Mb), tryptophan 2,3-dioxygenase (TDO), indoleamine dioxygenase 1 (IDO1), and heme oxygenase 2 (HO2) (19, 20, 21, 22, 23) and that heme insertion into many of these also requires the participation of the cell chaperone Hsp90 (22, 24, 25, 26, 27, 28). Accordingly, we investigated the potential for GAPDH and Hsp90 involvement in CYP3A4 and 2D6 heme allocation. We studied these CYP’s when they were transiently expressed in HEK293T, GlyA-CHO, and HEPG2 cells and when CYP3A4 expression was induced in HEPG2 cells by rifampicin, and we also investigated CYP associations with GAPDH and Hsp90 in cells under various conditions of CYP heme content. Our findings reveal that the primary means of mitochondrial heme allocation to CYP3A4 and 2D6 in cells is through a GAPDH- and Hsp90-driven mechanism. By identifying cellular processes for CYP3A4 and 2D6 heme allocation, our study sheds light on how these cytochromes mature to their functional forms and suggest new pathways that could regulate their activities in health or disease.

## Results

### Intracellular location of CYP3A4 and 2D6 in transfected HEK293T cells

**Figure 1:**
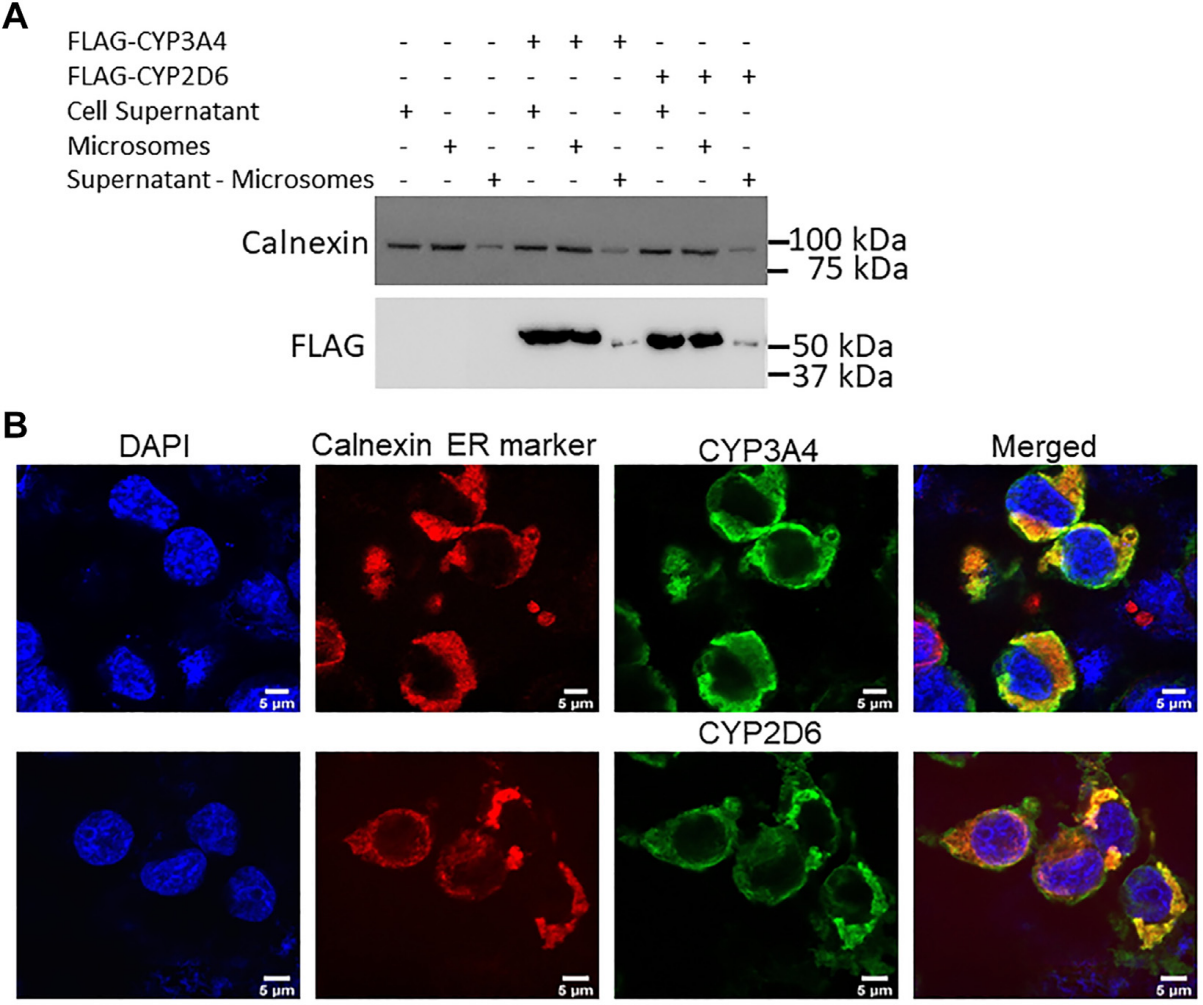
**Intracellular localization of CYP3A4 and 2D6 after transient expression in HEK293T cells.** FLAG and MYC-tagged CYP3A4 or 2D6 were transiently expressed in HEK293T cells. *A*, cell supernatant, microsomal fraction, and supernatant minus microsome samples (50 μg protein each) were run on SDS-PAGE followed by Western blot analysis for the presence of the ER protein Calnexin and for FLAG-tagged CYP in each sample. *B*, cells expressing either CYP3A4 or 2D6 protein were processed for confocal microsocopy as described in Experimental Procedures. The extent of Calnexin and CYP (FLAG) colocalization is indicated by the *orange* color in the merged panels.

We transfected HEK293T cells to transiently express FLAG and MYC-tagged human CYP3A4 or 2D6 and then examined their intracellular location by differential centrifugation of cell supernatants or by immunostaining of whole cell preparations. We found that both CYPs primarily localized to the microsomal-ER fraction in cell supernatants and in whole cells were primarily associated with the ER marker protein Calnexin (Figs. 1 and S1). These results are consistent with our CYP expression constructs containing their natural N-terminal ER anchoring sequences and confirm that their localization after transient transfection in HEK293T cells mimics their natural localization to the ER in liver (8, 10).

### Cell heme availability impacts the development of CYP activities but not their protein expression levels

**Figure 2:**
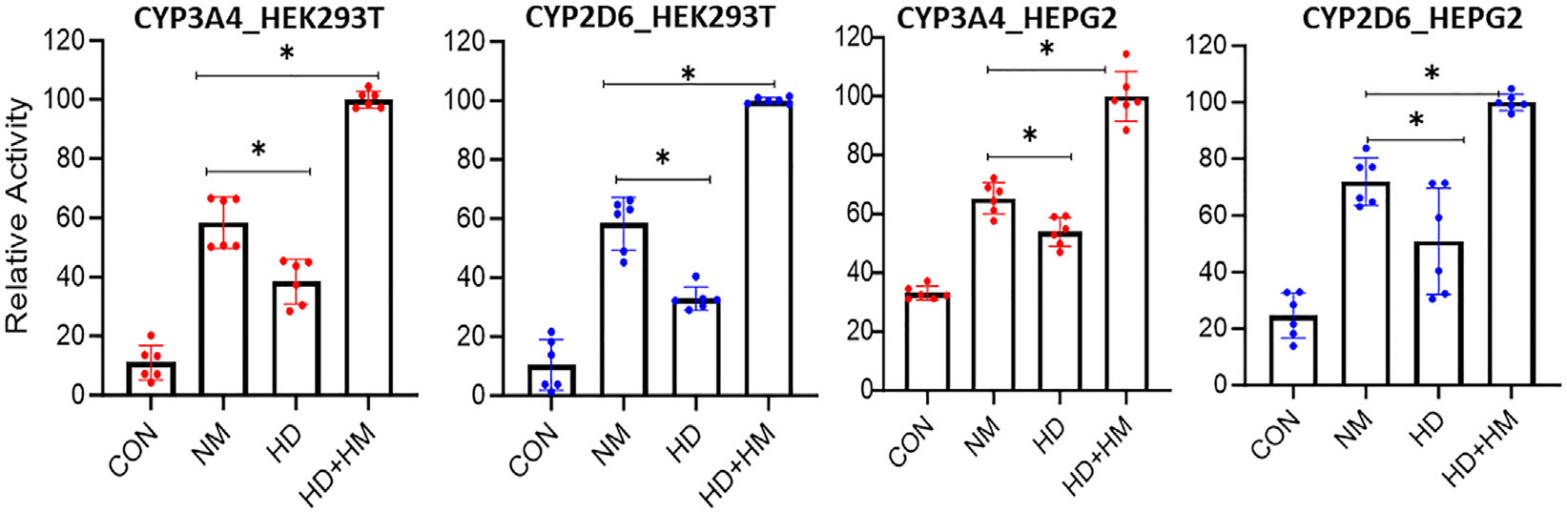
**Impact of cell heme availability on CYP activity.** HEK293T cells (*left* two panels) or HEPG2 cells (*right* two panels) were cultured either in normal media and serum (NM) or with medium containing SA and heme-depleted serum (HD) and were either transfected to express FLAG and MYC-tagged CYP 3A4 or 2D6 or were induced to express CYP3A4 by adding rifampicin (HEPG2, third panel). In some cultures, 5 μM hemin was added 3 h before cell harvest (HD + HM). After 36 h cells were harvested and CYP activities in the cell supernatants were measured. The values are from two independent experiments of three replicates each. Significance designation ∗*p* < 0.05, one-way ANOVA.

We next investigated how changes in cell heme availability might influence the CYP protein expression and activity levels. FLAG and MYC-tagged human CYP3A4 and 2D6 were transiently expressed in HEK293T cells under three conditions: In cells that were heme depleted due to prior culture with the heme biosynthesis inhibitor succinyl acetone (SA) (29) and heme-depleted serum (HD); in cells cultured with normal medium and serum (NM); and in cells given exogenous hemin (5 μM) for 3 h prior to harvest (HM). Controls included non-transfected cells that either did or did not receive the 5 μM hemin. The resulting CYP activities and expression levels, normalized to the total protein concentration of each cell supernatant sample, are shown in Figures. 2 and S2, respectively. Supernatants from non-transfected HEK293T cells had negligible activity as expected, whereas those from the transfected cells had activities that consistently differed according to the rank order of HM > NM > HD for both CYP3A4 and 2D6. The levels of CYP protein expression were not impacted by the differences in cell heme status (Fig. S2). Qualitatively similar results were obtained when HEPG2 cells that were cultured under the three different conditions of cell heme availability were either induced to express CYP3A4 by rifampicin treatment or were transfected to express CYP2D6 (Figs. 2 and S2). Thus, differences in cell heme availability similarly influenced CYP3A4 and 2D6 activities without altering their protein expression levels and did so in a way that implies the CYP’s were expressed as a mixture of their heme-replete and heme-free forms in cells that were grown under normal culture conditions.

### Functional maturation and heme allocation to CYP3A4 and 2D6 depend on the cell GAPDH expression level and its heme binding ability

Given that delivery of mitochondrial heme to several cytosolic hemeproteins is known to be GAPDH-dependent, we employed our previously established siRNA GAPDH knockdown and rescue strategies (19, 22) to examine if GAPDH is also involved in heme allocation to CYP3A4 and 2D6 and development of their activities. We performed siRNA knockdown of GAPDH expression in HEK293T cells cultured under normal conditions, and after 24 h these cells were transfected to express FLAG and MYC-tagged CYP3A4 or 2D6 alone or along with siRNA-resistant forms of HA-GAPDH wild type or the HA-GAPDH H53A variant that has defective heme binding but otherwise has normal glycolytic activity (19). After an additional 36 h of culture, the cell supernatants were prepared for analysis.

**Figure 3:**
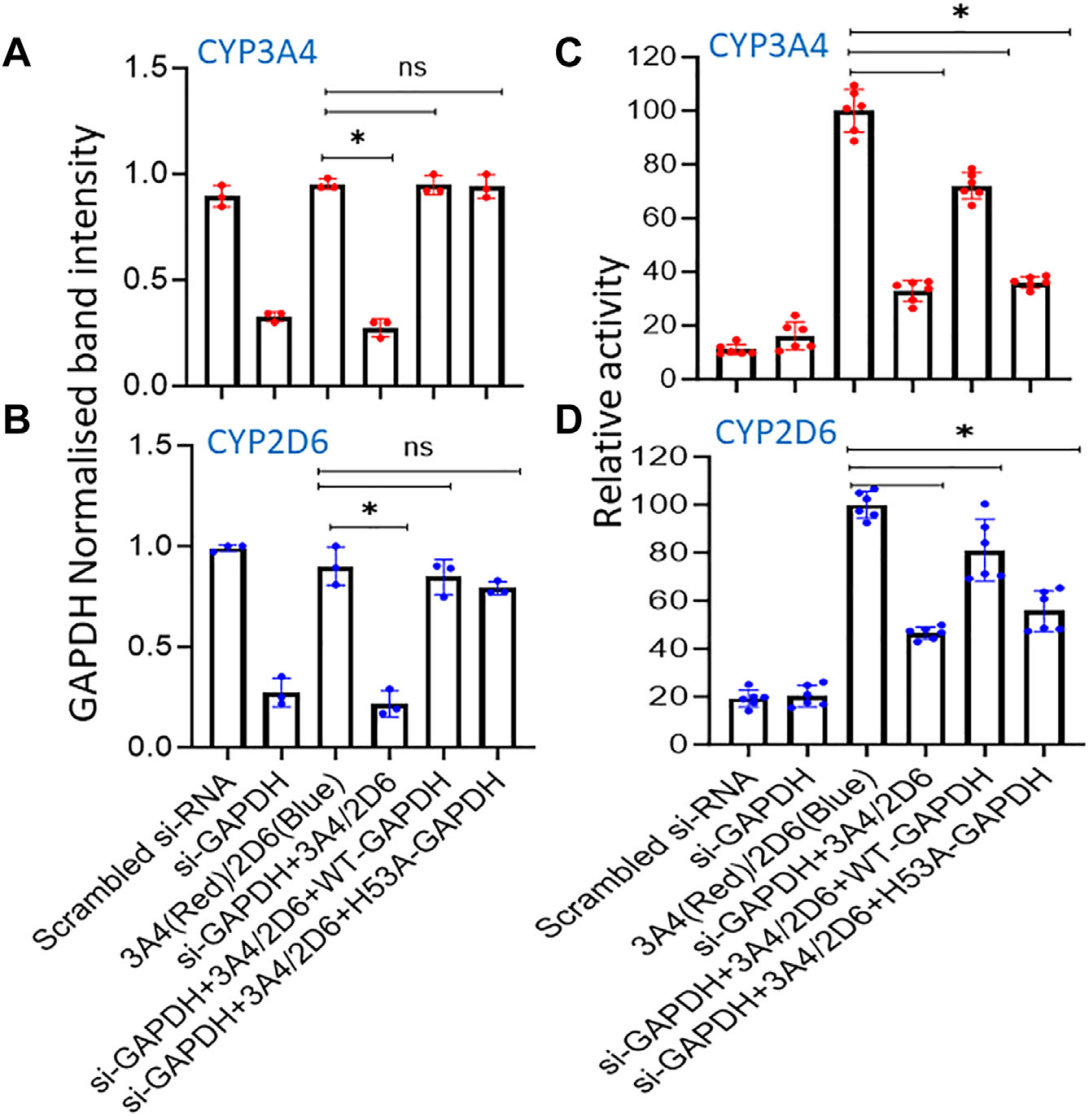
**GAPDH enables CYP3A4 and 2D6 maturation to function.** HEK293T cells that had been given scrambled or GAPDH-targeted siRNA were transfected to express FLAG and MYC-tagged CYP3A4 or 2D6 either alone or along with siRNA-resistant constructs of HA-tagged WT GAPDH or H53A GAPDH. *A* and *B*, quantification of the relative total GAPDH expression levels in the cells. *C* and *D*, CYP activities of the cell supernatants. Band intensities are from three independent experiments and the activity values are from two independent experiments of three replicates each. Significance designation ∗ *p* < 0.05, ns = not significant, one-way ANOVA.

The siRNA treatment lowered GAPDH expression in the HEK293T cells to approximately 20 to 30% the level of the scrambled siRNA control, and the expression of either HA-GAPDH protein in the knockdown cells recovered the total GAPDH expression levels to near normal (Fig. 3, *A* and *B*), all without impacting the expression levels of CYP3A4 or 2D6 (Fig. S3). However, the GAPDH knockdown caused the CYP3A4 and 2D6 activities to decrease to 18 and 39% of the positive control cell activity, respectively (Fig. 3, *C* and *D*). Co-expression of HA-GAPDH in the GAPDH knockdown cells significantly recovered the CYP3A4 and 2D6 activities, whereas co-expression of the H53A GAPDH variant did not (Fig. 3, *C* and *D*). These results indicate that the maturation of CYP3A4 and 2D6 catalytic activities largely depended on the cell GAPDH expression level and specifically on the heme binding function of GAPDH.

To determine if the GAPDH-dependence for CYP catalytic activities was related to heme allocation, we repeated the experiment using GlyA-CHO cells that had been made heme deficient and then underwent the siRNA-GAPDH knockdown procedure followed by transfection to express either FLAG and MYC-tagged CYP with and without the expression of the HA-tagged GAPDH wild type or H53A variant as explained earlier. The cells then had the heme precursors ^14^C-δ-ALA and ferric citrate added to promote mitochondrial ^14^C-heme generation, and after a further 6 to 8 h of culture, the cells were lysed and the supernatants were analyzed for CYP protein expression and for CYP ^14^C-heme incorporation after antibody pulldown.

**Figure 4:**
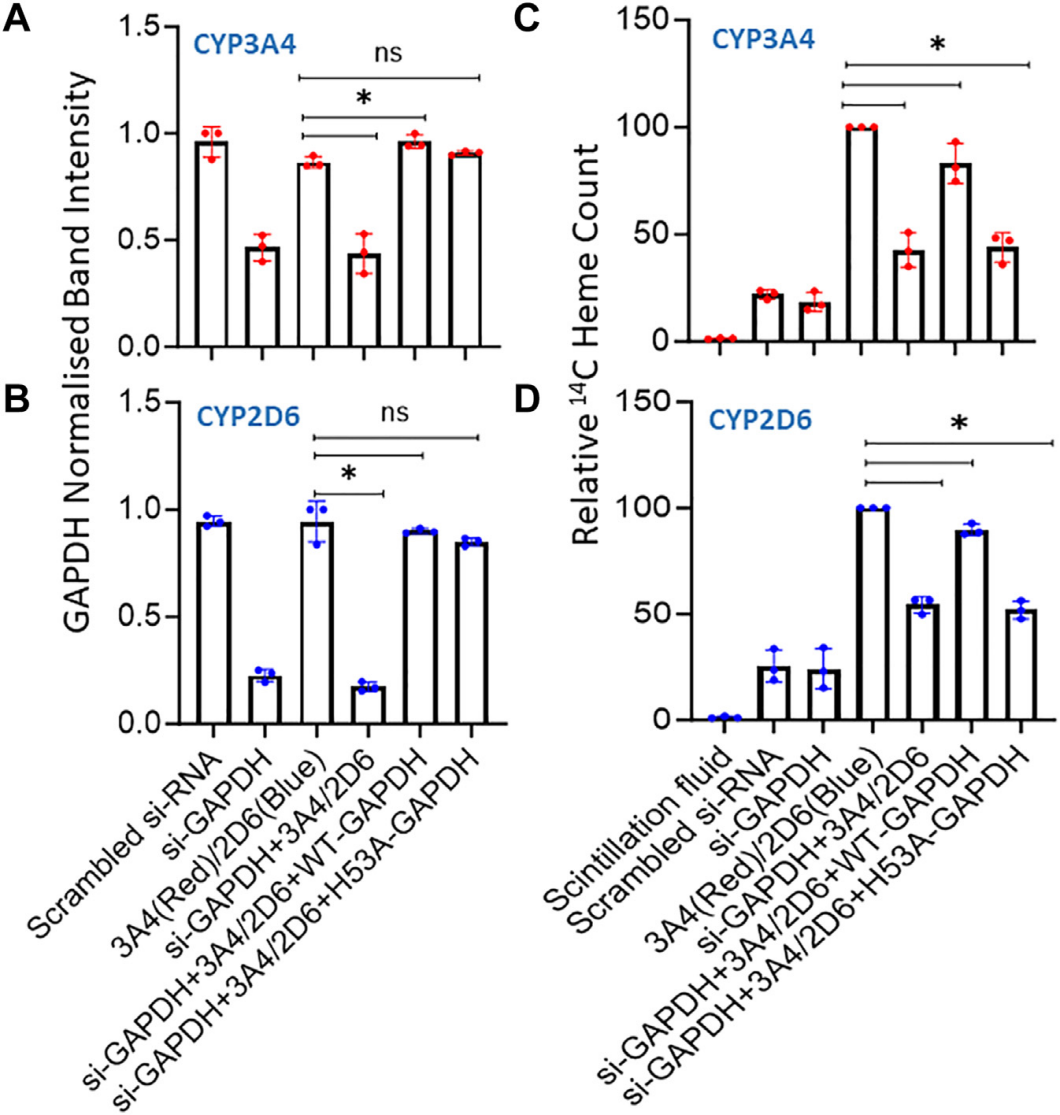
**GAPDH enables mitochondrial heme allocation to CYP3A4 and 2D6.** Heme-depleted GlyA-CHO cells underwent siRNA GAPDH knockdown or received scrambled siRNA and were then cultured as is or transfected to express FLAG and MYC-tagged CYP3A4 or 2D6 alone or along with HA-tagged wild type or H53A GAPDH. Cells were harvested for analysis 6 to 8 h after receiving the heme precursors ^14^C-δALA and Fe-cit. *A* and *B*, quantification of the relative total GAPDH expression levels. *C* and *D*, ^14^C counts in FLAG antibody pulldowns from each condition. Band intensities and ^14^C heme count values are from three independent experiments. Significance designation ∗ *p* < 0.05, ns = not significant, one-way ANOVA.

Figure 4, *A* and *B* show that the siRNA knockdown treatment lowered the GAPDH expression level in the GlyA-CHO cells to approximately 30 to 50% of the level in the scrambled siRNA control cells and show that transfection of the knockdown cells to express either HA-GAPDH wild type or the H53A GAPDH variant brought their total GAPDH expression levels back to near normal, with these manipulations not significantly impacting the level of CYP protein expression (Fig. S4). However, cells that underwent the GAPDH knockdown had only 40% and 50% ^14^C-heme content in their CYP3A4 and 2D6 relative to the positive control, respectively (Fig. 4, *C* and *D*). Their ^14^C-heme levels recovered to reach 75 and 85% of the positive control, respectively, when the GAPDH knockdown cells co-expressed the HA-GAPDH wild type but did not recover in cells that co-expressed the H53A GAPDH variant (Fig. 4, *C* and *D*). These differences in CYP ^14^C-heme incorporation matched with the differences we observed for the CYP catalytic activity measures as described in Fig. 3. Together, our findings reveal that CYP3A4 and 2D6 rely on GAPDH allocation of mitochondrial heme to mature to catalytic function in the cells.

### Functional maturation of CYPs and their heme incorporation are Hsp90-dependent

**Figure 5:**
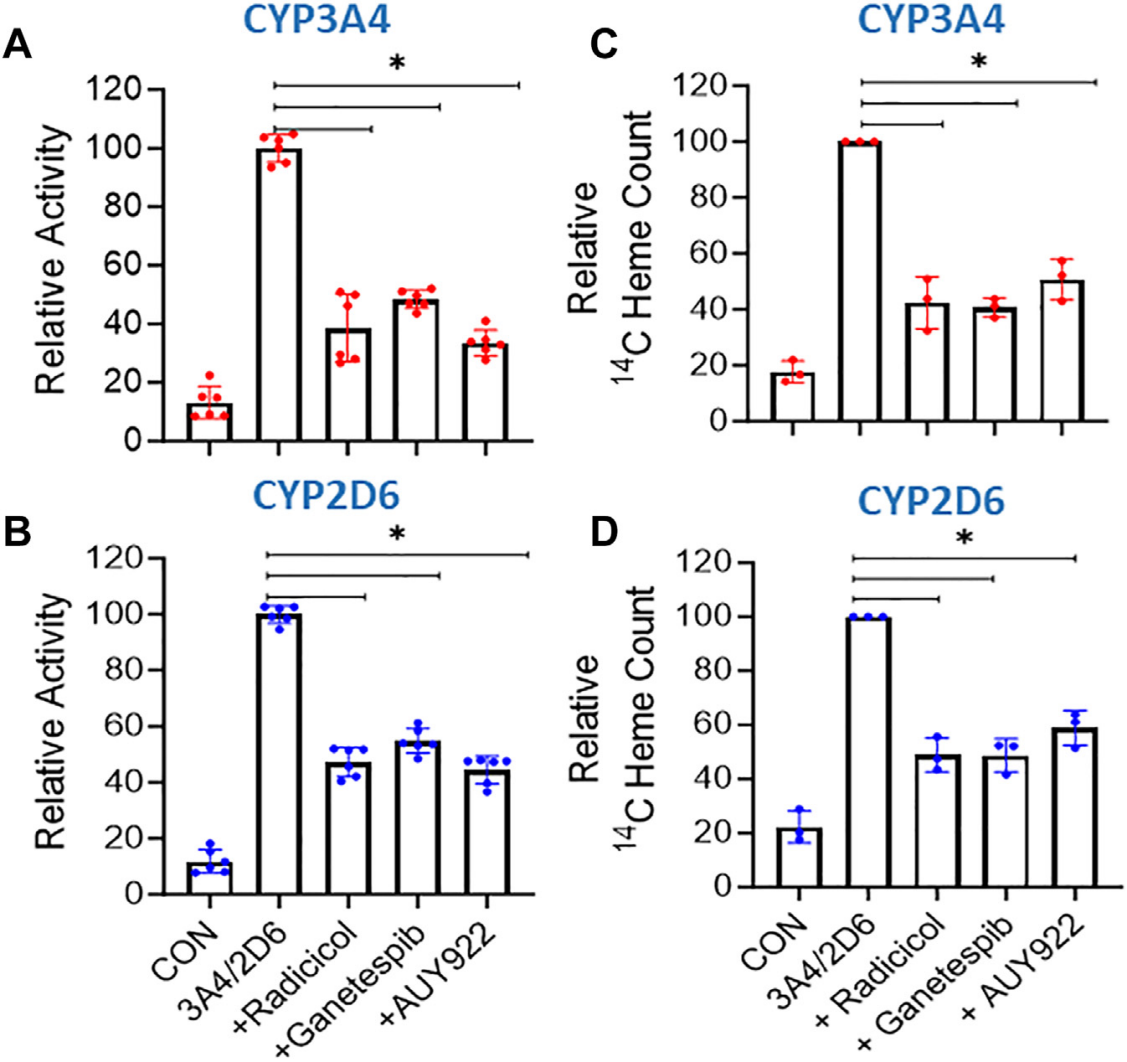
**Effect of Hsp90 inhibitors on the functional maturation and heme content of CYP3A4 and 2D6.** Heme-depleted HEK293T or GlyA-CHO cells were transfected to express FLAG- and MYC-tagged CYP3A4 or 2D6 and then given heme precursors without or with the Hsp90 inhibitors radicicol, ganetespib, or AUY922, and then cells were harvested after a further 6 to 8 h of culture. *A* and *B*, CYP activities measured in HEK293T cell supernatants for each condition. *C* and *D*, ^14^C counts in CYP antibody pulldowns from GlyA-CHO cell supernatants for each condition. Activity values are from two independent experiments of three replicates each. ^14^C heme count values are from three independent experiments. Significance designation ∗ *p* < 0.05, ns = not significant, one-way ANOVA.

The cell chaperone Hsp90 is required to drive heme insertion into several different cytosolic heme proteins (28). To test if Hsp90 plays a similar role in CYP3A4 and 2D6 maturation and heme allocation, we examined how each of the three Hsp90 inhibitors (Radicicol, AUY922, and Ganetespib) would impact the ability of the mitochondrial heme precursors δ-ALA and Fe-citrate to increase CYP catalytic activities. Following CYP expression in heme-deficient HEK293T cells, they received δ-ALA + Fe-citrate along with buffer alone or containing radicicol (10 μM), AUY922 (5 μM), or Ganetespib (500 nM), and after a further 6 to 8 h culture, the cells were lysed for analysis. The individual impact of each Hsp90 inhibitor was similar and they diminished development of CYP3A4 and 2D6 activities to approximately 25% and 47% of the positive control group, respectively (Fig. 5, *A* and *B*), without altering the CYP protein expression levels (Fig. S5).

To investigate if the Hsp90 inhibitors acted by antagonizing heme incorporation into the CYPs, we repeated the experiments as described above using GlyA-CHO cells and ^14^C-δ-ALA plus Fe-citrate so that the cells would generate ^14^C-heme, and then measured its incorporation into the FLAG and MYC-tagged CYP proteins after antibody pulldown from the supernatants. Figure 5, *C* and *D* show that in the presence of each Hsp90 inhibitor the incorporation of mitochondrial-generated ^14^C-heme into CYP3A4 and 2D6 was diminished to approximately 25% and 40% the level of the positive control, without altering the expression level of either CYP (Fig. S5). We conclude that each Hsp90 inhibitor significantly diminished functional maturation of the CYP’s by blocking insertion of mitochondrial heme into their apo-protein forms.

### Impact of combined GAPDH knockdown and Hsp90 inhibition on CYP3A4 heme allocation

**Figure 6:**
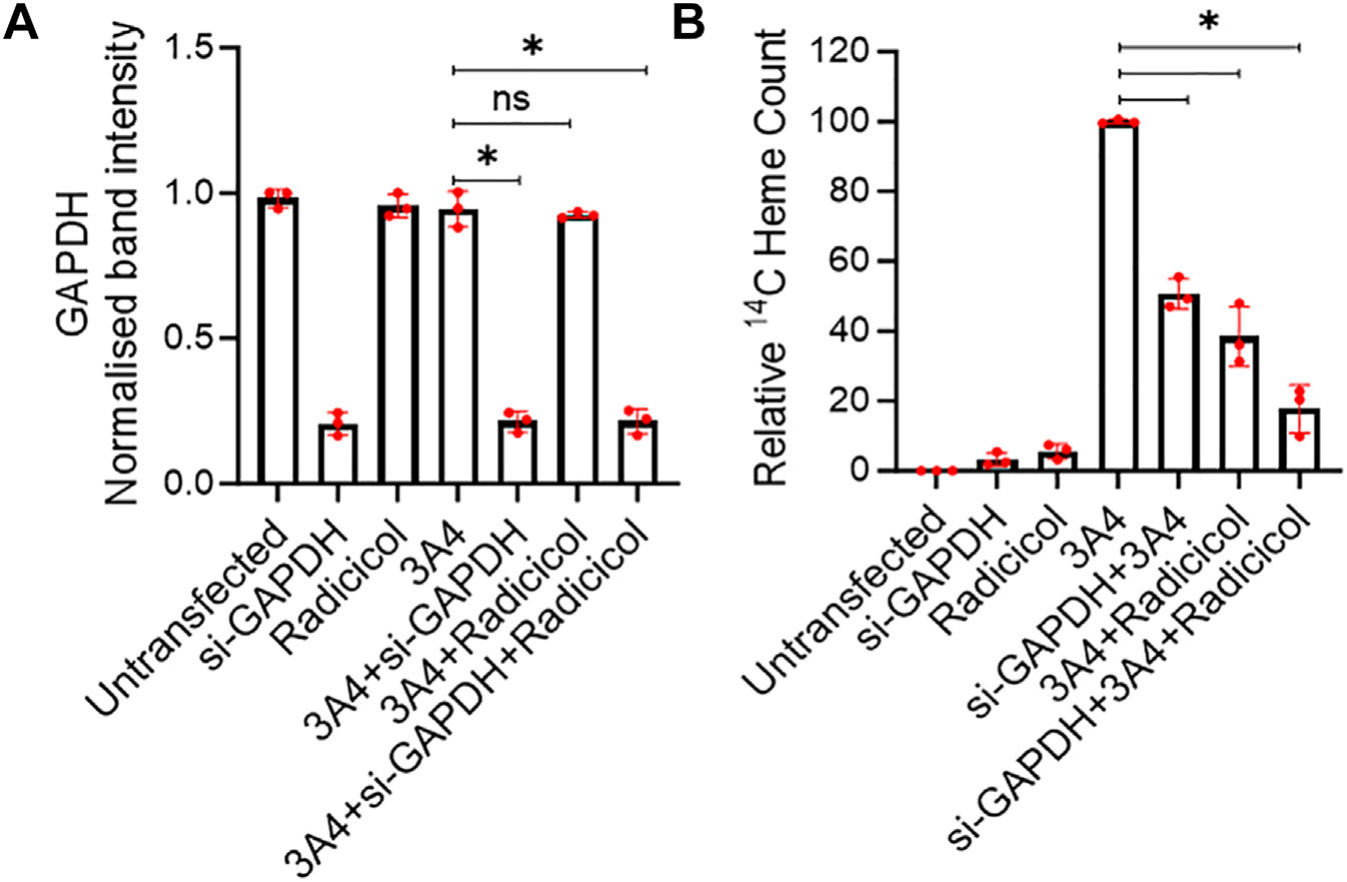
**Combined GAPDH knockdown and Hsp90 inhibition has an additive effect.** Heme-depleted GlyA-CHO cells underwent siRNA GAPDH knockdown as indicated. After 24 h, the plates were re-transfected to express FLAG and MYC-tagged CYP3A4. After 30 h the plates were treated with 10 μM Radicicol and the cells were harvested after a further 6 to 8 h of culture. In some cases, cells were not transfected to express CYP3A4, and/or were given only the scrambled siRNA, siGAPDH, or radicicol treatment alone. Cell supernatants underwent SDS-PAGE and Western blotting to assay protein expression levels or antibody pulldown to determine ^14^C heme counts in CYP3A4. *A*, Comparative GAPDH expression levels. *B*, Relative ^14^C heme counts in the CYP pulldowns. Results are the mean ± SD of three replicates. Significance designation ∗ *p* < 0.05, ns = not significant, one-way ANOVA.

We next performed an experiment to determine how a combined knockdown of GAPDH expression and Hsp90 inhibition would impact cell CYP heme allocation. GlyA-CHO cells underwent siRNA GAPDH knockdown or received scrambled siRNA and the plates were transfected to express FLAG and MYC-tagged CYP3A4. The cells were then given ^14^C-δ-ALA and Fe-citrate and treated with vehicle or 10 μM Radicicol and harvested after 6 to 8 h. Figure 6*A* shows the siRNA silencing led to an 80% reduction in GAPDH expression in all cases. GAPDH knockdown alone led to a 50% lower ^14^C heme level in the CYP3A4 relative to the positive control (Fig. 6*B*), and radicicol treatment alone led to a 60% lower ^14^C heme level. When the two treatments were combined it led to an 85% lower ^14^C heme level in CYP3A4. None of the treatments altered the cell CYP3A4 expression level (Fig. S6). Together the results suggest that combined GAPDH knockdown and hsp90 inhibition have an additive negative impact on cell heme allocation to CYP3A4.

### Intracellular associations between CYP’s and Hsp90 or GAPDH

**Figure 7:**
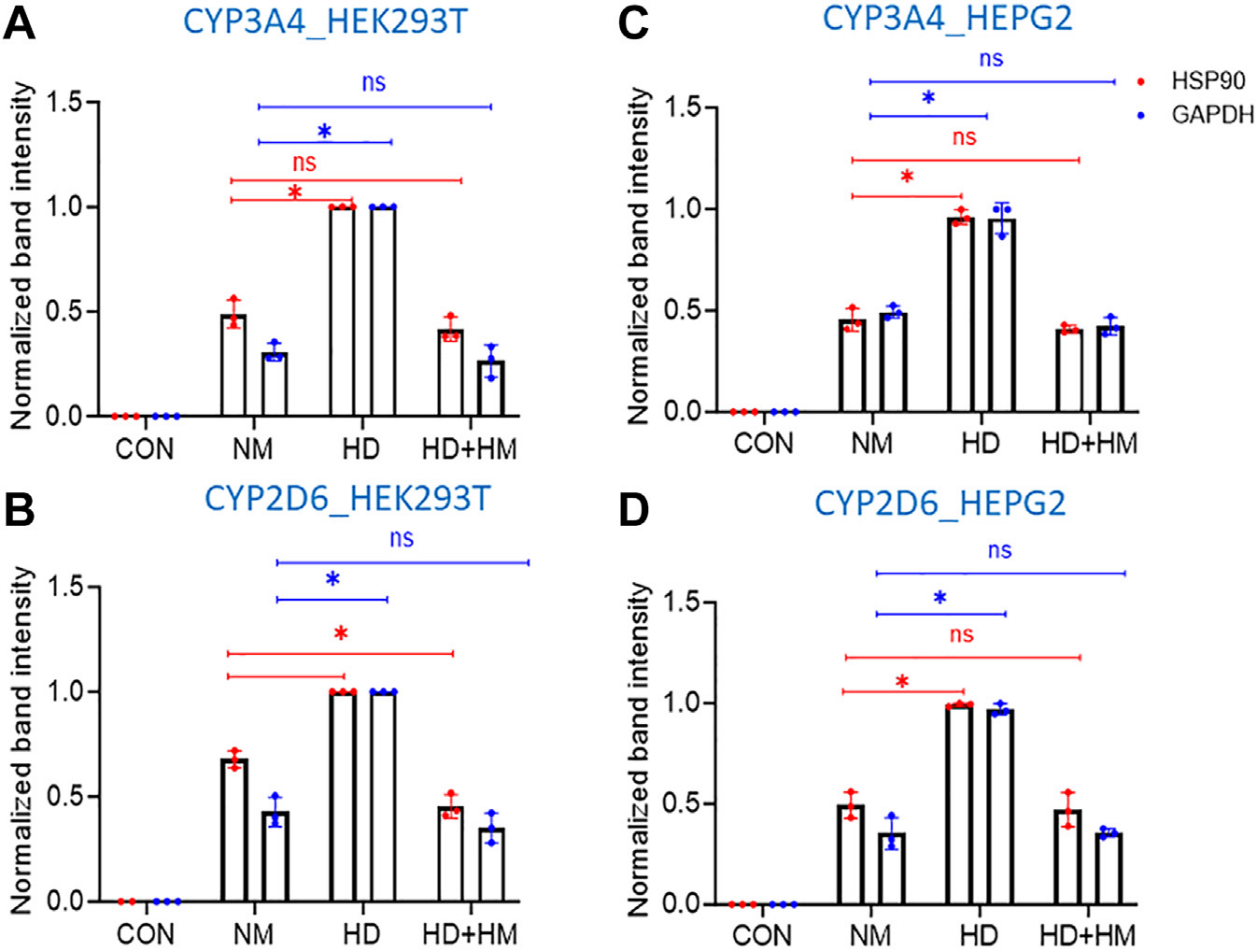
**Impact of different cell heme levels on the intracellular association of CYP3A4 or 2D6 with GAPDH or Hsp90.** HEK293T cells (*Panels A* and *B*) or HEPG2 cells (*Panels C* and *D*) were cultured either in normal media and serum (NM) or with medium containing SA and heme-depleted serum (HD) and were transfected to express FLAG and MYC-tagged CYP 3A4 or 2D6. In some cultures, hemin was added 3 h before cell harvest (HD + HM). After 36 h cells were harvested and the supernatants underwent FLAG Ab pulldown. The levels of GAPDH and Hsp90 association were determined by SDS-PAGE and Western blotting and were normalized to the levels of the CYP3A4 or 2D6 bands present in the pulldowns. Values are from three independent experiments. Significance designation ∗ *p* < 0.05, ns = not significant, one-way ANOVA.

We next investigated if the CYP proteins associate with Hsp90 or GAPDH in cells and if their associations would be influenced by CYP heme status. The FLAG and MYC-tagged CYP3A4 or 2D6 were expressed in HEK293T cells that were either heme-deficient, cultured normally, or cultured with added hemin as described before. Figures 7, *A*, *B* and S7 upper panels, show that upon pulldown with an anti-FLAG antibody the CYP3A4 and 2D6 were each associated with Hsp90 and with GAPDH under all three conditions of cell culture but were most heavily associated with Hsp90 and GAPDH in the heme-deficient cells. Similar results were observed when rifampicin was used to induce HEPG2 cells to express CYP3A4 or when HEPG2 cells were transfected to express CYP2D6 (Figs. 7, *C*, *D* and S7 lower panels). We also found that GAPDH and Hsp90 were associated with the CYP3A4 and 2D6 proteins naturally expressed in mouse liver (Fig. S8). Together, this shows that CYP3A4 and 2D6 associate with GAPDH and Hsp90 in cells to an extent that is inversely related to their heme contents.

### Cell heme allocation to CYP3A4 and 2D6 is independent of PGRMC2

**Figure 8:**
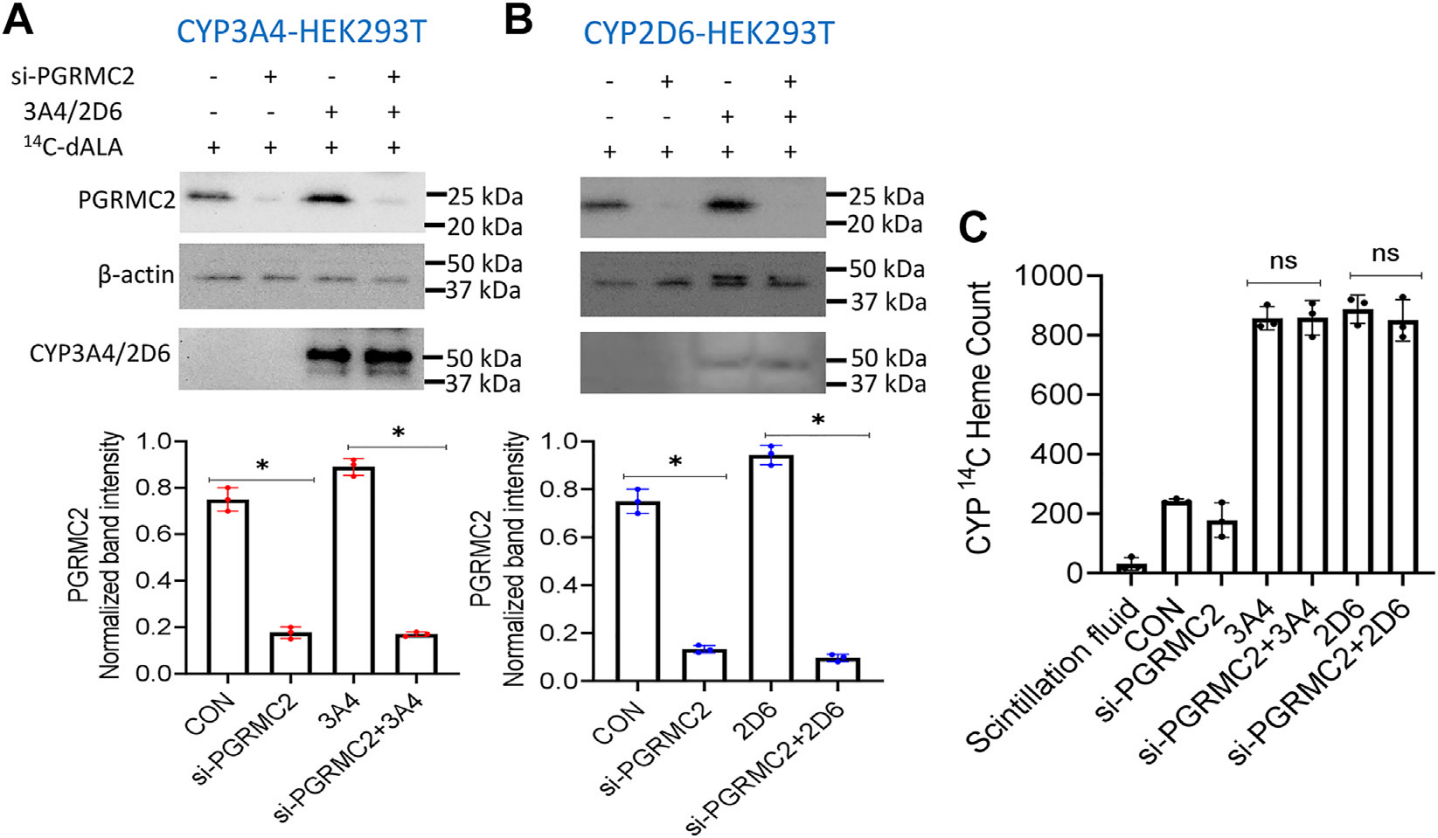
**Impact of PGRMC2 knockdown on cell heme allocation to CYP3A4 and 2D6.** HEK293T cells were transfected with PGRMC2-directed siRNA for 24 h or given media alone and then were transfected to express FLAG and MYC-tagged CYP3A4 or 2D6 along with concurrent provision of ^14^C-δ-ALA + Fe-citrate, followed by harvest after an additional 8 h of culture. *A* and *B*, Representative Western blots indicating the relative expression levels of PGRMC2, CYP’s, and β-actin, and quantitative plots comparing the level of PGRMC2 expression under the various conditions. *C*, ^14^C counts in FLAG Ab pulldowns from supernatants of cells cultured under the indicated conditions. Band intensity and ^14^C-heme count values are from three independent experiments. Significance designation ∗ *p* < 0.05, ns = not significant, one-way ANOVA.

Because the ER protein PGRMC2 was recently implicated in intracellular heme transport (28) and our CYP3A4 and 2D6 proteins were primarily ER-localized, we tested for its possible involvement in our system. HEK293T cells underwent a 24 h treatment with PGRMC2 siRNA or vehicle and were then transfected to express FLAG and MYC tagged CYP3A4 or 2D6 for 30 h and were also given ^14^C-δ-ALA and Fe-citrate and cultured for an additional 7 to 8 h prior to lysis. The supernatants were subject to pulldown and Western blot analyses. The siRNA treatment caused 80 to 85% loss in PGRMC2 expression in the cells but this had no impact on CYP protein expression level (Fig. 8, *A* and *B*) or on the level of CYP ^14^C-heme incorporation (Fig. 8*C*). Thus, PGRMC2 is not likely to be involved in mitochondrial heme allocation to CYP3A4 or 2D6 in our system.

## Discussion

Given that mitochondrial heme allocation is required for CYP’s to mature to function, an understanding of this process is fundamentally important. Our study reveals that the allocation of mitochondrially generated heme to human CYP3A4 and 2D6 in mammalian cells substantially relies on the expression level and the heme-binding ability of GAPDH and the activity of chaperone Hsp90. This was demonstrated in three types of cells that expressed CYP3A4 or 2D6 following transient transfection and was observed when cells were cultured in conditions of normal heme availability or were cultured to be heme-depleted and then subsequently stimulated to generate mitochondrial heme from δ-ALA and Fe-citrate. Thus, CYP3A4 and 2D6 join a growing list of heme proteins whose heme allocations depend on GAPDH and in most cases on Hsp90, which presently includes the inducible and neuronal NO synthases, sGCβ, IDO1, TDO (independent of Hsp90), HO2, and the globins Hb-β, Hb-γ, and Mb (19, 20, 21, 22, 23, 24, 25, 26, 27, 28, 30).

Our pulldown experiments showed that GAPDH and Hsp90 were associated with CYP3A4 and 2D6 in cells. This is consistent with the CYP proteins being ER-anchored *via* their N-terminals such that some of their protein structures remain exposed to the cell cytosol and thus able to interact with cytosolic protein partners like GAPDH or Hsp90 (10, 11). The degree of GAPDH or Hsp90 association with CYP3A4 or 2D6 appeared to be inversely related to their heme levels (*i.e.*, a greater CYP heme content correlated with a lesser protein partner association). This inverse relationship has also been observed for other GAPDH- and Hsp90-dependent heme proteins and suggests that CYP associations with GAPDH and Hsp90 are likely important for heme allocation and that CYP affinities toward these proteins diminish after the heme insertion takes place. These findings provide a rationale to further investigate their direct interactions and mechanisms that enable CYP heme delivery.

Our findings with CYP3A4 and 2D6 are consistent with heme delivery to another ER-associated protein, HO2, being GAPDH-dependent (23), and thus provide additional evidence that GAPDH heme allocations are not strictly limited to heme proteins that mature and reside in the cytosol. However, our current findings indicate that cell heme allocations to CYP3A4 and 2D6 were substantially but not completely dependent on GAPDH or Hsp90, given that between 25 to 50% of heme delivery still occurred in cells that either underwent the GAPDH knockdown or the pharmacologic Hsp90 inhibition. For GAPDH this might reflect our achieving only a partial (50–80%) knockdown of the GAPDH expression level in the cells. Indeed, in cells that underwent a combined GAPDH knockdown and Hsp90 inhibition, there appeared to be an additive effect that led to greater inhibition of CYP heme allocation. In any case, our current findings suggest the possibility that additional heme delivery pathways may be available for these CYP’s. Although our results argue against participation by the ER protein PGRMC2, other pathways such as heme delivery through mitochondria-ER membrane contacts remain to be explored (18).

The process of intracellular heme delivery and insertion into CYP3A4 and 2D6 is highly relevant for their biological function because they both depend on an incorporated heme to catalyze oxidations that activate, inactivate, and/or help to detoxify and eliminate a broad range of drugs and xenobiotic compounds. Thus, our findings imply that anything that would positively or negatively impact GAPDH- and Hsp90-dependent heme allocation in cells would influence the biological activities of CYP3A4 and 2D6 and thereby impact their contributions to drug or xenobiotic metabolism. For example, it is known that higher levels of NO exposure inhibit cells from inserting heme into CYP3A4 and 2D6 (31) and can also inhibit GAPDH-dependent heme delivery (32, 33). Thus, it is conceivable that the elevated NO levels achieved during inflammation may help suppress CYP3A4 and 2D6 activities by impacting their GAPDH-dependent heme allocation. This would present a new mechanism whereby NO can inhibit CYP functions during inflammation (34). Similar concerns hold regarding the impact of chronic Hsp90 inhibition, given that Hsp90 is a target in anti-cancer pharmaceutical development and clinical trials (35, 36). These potentially new modes of regulating CYP3A4 and 2D6 maturation and activity *via* control of their cell heme allocations can now be explored.

## Conclusion

Identifying roles for GAPDH and Hsp90 in heme allocation to human CYP3A4 and 2D6 sheds new light on their cellular maturation processes and reveals new potential points of physiologic or pharmacologic control that could regulate their activities in drug metabolism, inflammation, cancer, or other diseases. Several questions arise from our study: Do other members of the CYP family show a similar or different degree of dependency on GAPDH and Hsp90 for their heme allocations, and if so, which ones? How do GAPDH and Hsp90 function at the molecular level to enable heme allocation to the CYP’s? Is heme allocation to CYP’s altered in inflammatory settings or in other diseases to a point that impacts their biological functions? These questions have broad implications for CYP enzyme function in biology and are worthy of further investigation.

## Experimental procedures

### Reagents

Human FLAG and MYC-tagged CYP3A4 (RC210170) & CYP2D6 (RC223749) expression plasmids were purchased from Origene. Hsp90 inhibitor Radicicol (R2146), heme biosynthesis inhibitor succinyl acetone (D-1415), protein synthesis inhibitor (cycloheximide #C7698), hemin chloride (3741), sodium dithionite (157953), ferric citrate (F3388) and rifampicin (SIAL-R3501) were all purchased from Sigma. Hsp90 inhibitor Ganetespib (HY-15205) and AUY-922 (N-5300) were purchased from MedChem Express & LC labs respectively. HEK293T (CRL-11268) and HEPG2 cells were purchased from the American Type Culture Collection. GlyA-CHO cells were a gift from Dr P. J. Stover, Cornell University. Cell culture media DMEM (11-500), Ham’s F12K (37-500CUST), and EMEM (99BJ500CUST) were purchased from the Cleveland Clinic media core. Fetal Bovine Serum (10437028) was purchased from Gibco. si-RNA against human GAPDH (D-001830-01-20) and accompanying scrambled si-RNA (D-001810-10-05) were purchased from Horizon discoveries, si-RNA against human PGRMC2 (sc-88944) was purchased from Santa Cruz Biotechnology. Lipofectamine 2000 was purchased from Invitrogen (11668019). Anti-FLAG (F1804) & anti-β-actin (A5441) antibodies were purchased from Sigma. Anti-GAPDH (14C10), anti-Hsp90 (4874), anti-CYP3A4 (13384) & anti-CYP2D6 (73867) antibodies were purchased from Cell Signaling Technologies. Anti-PGRMC2 antibody was purchased from Santa Cruz Biotechnology (sc-374624). Anti-mouse (170-6516) and anti-rabbit (170-6515) antibodies were purchased from Bio-Rad. ECL substrate (32106) was from Thermo Scientific. ^14^C labeled δ-Amino levulinic acid (δ-ALA) was purchased from ChemDepo Inc. CYP3A4 & 2D6 activity assay kits were purchased from Abcam (ab211076 & ab211078). Protein G Sepharose fast flow beads (17061801) were purchased from Cytiva. PVDF membrane (1620177) & blocking grade milk (1706404) were purchased from Bio-Rad. Protease inhibitor cocktail (EDTA free, 11873580001) was purchased from Roche.

### CYP expression in HEK293T cells

HEK293T cells were grown in 10 cm plates in DMEM media with normal FBS (NM) or with heme-depleted FBS (HD) and 400 μM succinyl acetone (SA) for 72 h. Cells were transfected with plasmids coding for FLAG-MYC tagged human CYP3A4 or CYP2D6 (10 μg/plate) using Lipofectamine 2000. In some of the transfected plates containing HD serum and SA, the media was changed to remove SA and 5 μM hemin was added. At 36 h after transfection, the cells were washed with cold PBS and cold lysis buffer (40 mM EPPS, 150 mM NaCl, 10% glycerol, pH 7.6) containing protease inhibitors. The cells were then harvested and underwent five freeze-thaw cycles, were centrifuged at 10,000*g* for 20 min, and the supernatants were collected and stored at −80 °C until use.

### CYP expression in GlyA-CHO cells, ^14^C labeled heme production

GlyA-CHO cells were grown for 3 days in Kaighn's modified medium with heme-depleted serum and 400 μM of SA in 10 cm plates. Once the cells reached 70% confluency, they were transfected with 10 μg of FLAG and MYC-tagged CYP3A4 or 2D6 expression plasmids using Lipofectamine 2000. After 30 h of culture, the media in each plate was exchanged for 5 ml of glycine-deficient F12K media containing heme-depleted serum with SA. After 1 h, Fe-cit (25 μM) and ^14^C-labeled δ-ALA (3.5 μCi, 14 μM final) were added. The cells underwent an additional 7 to 8 h of culture and then were washed with PBS at 4 °C and harvested using cold lysis buffer (40 mM EPPS, 150 mM NaCl, 10% glycerol, pH 7.6) containing protease inhibitors. The cell suspensions underwent five freeze-thaw cycles, were centrifuged at 10,000*g* for 20 min, and the supernatants were collected and stored at −80 °C until use.

### Expression of CYP3A4 or 2D6 in HEPG2 cells

HEPG2 cells were cultured in EMEM with 10% normal or heme-depleted FBS and with SA (400 μM). CYP3A4 expression was induced using 50 μM of rifampicin for 48 h. In some plates, 5 μM hemin was added 3 h before cell harvest. Since there was no detectable induction of CYP2D6 in the HEPG2 cells in response to rifampicin, they were instead transfected with 10 μg of FLAG-MYC tagged CYP2D6 plasmid as was described above for the HEK293T and GlyA-CHO cells.

### Transfection of siRNA and gene silencing

HEK293T or GlyA-CHO cells were grown in 10 cm plates. The cells were transfected at 60 percent confluency with commercially available siRNA against human GAPDH, human PGRMC2, or scrambled siRNAs (75 nM per 10 cm plate) using Lipofectamine 2000. After 24 h the cells were re-transfected with 10 μg of FLAG and MYC-tagged CYP3A4 or CYP2D6 or empty vector. After 36 h of culture, the plates were washed with ice-cold PBS and given cold lysis buffer (40 mM EPPS, 150 mM NaCl, 10% glycerol, pH 7.6) containing protease inhibitors. The cells were then harvested and underwent five freeze-thaw cycles, were centrifuged at 10,000*g* for 20 min, and the supernatants were collected and stored at −80 °C until use.

### CYP3A4 and 2D6 activity assays

The CYP3A4 and 2D6 activities in cell supernatants were measured using fluorometric assay kits from Abcam (ab211076 & ab211078). The kit protocol was followed to perform activity assays on approximately 50 μg of supernatant protein in a final volume of 70 μl in each well. The fluorescent change kinetics were measured for 45 min at 37 °C on a microplate reader using Ex/Em = 535/587 nm for CYP3A4 and 60 min at Ex/Em = 390/468 nm for CYP2D6. Activities were calculated from the slopes and a standard plot using resorufin for CYP3A4 and AHMC for CYP2D6 and are reported as picomole/min/μg of protein.

### Immunoprecipitation of proteins on beads

Cell supernatant (1 mg protein) was mixed with 3 μg of antibodies directed against FLAG, CYP3A4 or 2D6, or GAPDH, mixed on a rotor at 4 °C for 1 h, then 20 μl of protein G-Sepharose beads (Cytiva) were added and the mixture was left rotating overnight at 4 °C. The beads were then centrifuged at 700*g* and washed 3× with cold 40 mM EPPS, 150 mM NaCl, 10% glycerol, 0.5% Nonidet P-40, pH 7.6. The washed beads were either boiled with Laemmli buffer, centrifuged, and the solution loaded onto SDS- PAGE gels, or were directly mixed with scintillation fluid for ^14^C radioactive heme counting.

### Western blotting

Cell supernatants and bead suspensions were resolved onto 8.5% SDS-PAGE and then transferred to PVDF membranes. The membranes were blocked using 5% of non-fat blocking grade milk for 30 min and then primary antibodies against the following were added: FLAG (dilution 1:1000), CYP3A4 or CYP2D6 (dilution 1:1000), Hsp90β (dilution 1:1000), GAPDH (1:2500), β-Actin (dilution 1:2500) and PGRMC2 (1:200). The proteins were detected by chemiluminescence using HRP-conjugated secondary antibodies of either anti-mouse (dilution 1:5000) or anti-rabbit (dilution 1:5000) origin and ECL substrate. The images were acquired using a chemidoc system from Bio-Rad and band intensities on the Western blots were quantified using Image J software (NIH).

### Microsome isolation

Microsomes were isolated using a Microsome Isolation Kit (Catalog Number MAK340) according to the kit protocol. HEK293T cells were transfected with FLAG and MYC-tagged CYP3A4 or 2D6 and after 40 h the cells were detached using trypsin/EDTA, washed with ice-cold PBS, and centrifuged at 700*g* for 5 min. The cells were resuspended in a cold homogenization buffer and transferred to a chilled Dounce homogenizer and homogenized. The homogenate was transferred to a microcentrifuge tube, vortexed for 30 s, and then centrifuged at 10,000*g* for 15 min at 4 °C. The floating lipid layer was aspirated off, a portion of the supernatant was saved, and the remaining supernatant was centrifuged at 20,000*g* for 20 min at 4 °C. The floating layer was aspirated off, a portion of the supernatant was saved, and the light beige/pink pellet was gently washed with homogenization buffer and resuspended in ice-cold storage buffer. Protein concentrations were measured and the supernatant and microsome samples were stored at −80 °C until use.

### Mouse liver homogenization

A sample of fresh mouse liver was washed 3× with cold PBS and then was minced in 500 μl of cold PBS, centrifuged at 6000*g* and the supernatant was stored at −80 °C. Protein concentration was measured using the Bradford assay.

### Microscopic imaging

HEK293T cells were grown under normal conditions on glass coverslips immersed in six-well tissue culture plates and were transfected to express FLAG- and MYC-tagged CYP3A4 or 2D6. After transfection for 36 to 40 h, cells on the coverslips were fixed with 4% formaldehyde in PBS, washed with PBS, and then permeabilized with 0.3% Triton X-100 in PBS. Following blocking with 1% BSA the coverslips underwent overnight incubation with primary antibodies (anti-FLAG) and Calnexin (Thermofisher Scientific, PA5-34754). Secondary antibodies were added after rinsing and incubated for 1 h at RT. Coverslips were washed 3× for 5 min under gentle agitation and were mounted onto glass slides using *in situ* mounting medium containing DAPI, then dried and sealed. The slides were imaged using a confocal microscope with an objective lens of 100×.

### Data and statistical analyses

SDS-PAGE gels of cell supernatants had equal total protein loaded per lane. Following Western blotting, the measured band intensity of the protein of interest (GAPDH, CYP, PGRMC2, Hsp90) was normalized by the sample’s corresponding β-actin band intensity. The resulting values were graphed on a relative basis. The CYP activities of each sample were reported as per mg of supernatant protein. The activity values within each trial were normalized based on the sample with the highest activity and then graphed on that relative basis. The ^14^C heme counts were derived from antibody pulldowns done with equal amounts of supernatant protein for each sample. Count values within each trial were normalized based on the sample with the highest counts and then were graphed on that relative basis. The statistical test used to measure the significance (*p*-values) between two groups was one-way ANOVA in the software Graph Pad Prism (v9).

## Data availability

All data are contained within this manuscript or are available from the authors.

## Supporting information

This article contains supporting information.

## Conflict of interest

The authors declare that they have no conflicts of interest regarding the contents of the article.
