# Supplementary material for: Functional maturation of cytochromes P450 3A4 and 2D6 relies on GAPDH- and Hsp90-Dependent heme allocation
Source: J Biol Chem. 2024 Jan 8;300(2):105633. doi: 10.1016/j.jbc.2024.105633 (PMC10840333; doi:10.1016/j.jbc.2024.105633)

Functional Maturation of Cytochromes P4503A4 and 2D6 Relies on GAPDH- and Hsp90-Dependent Heme Allocation

Sidra Islam, Dhanya Thamaraparambil Jayaram, Pranjal Biswas, and Dennis J. Stuehr*

Figure Number	Page Number
S1	2
S2	3
S3	4
S4	5
S5	6
S6	7
S7	8
S8	9

Figure S1. Confocal images of HEK293T cells not expressing CYP proteins that were treated with DAPI or the fluorescent labeled antibodies directed against the indicated proteins.

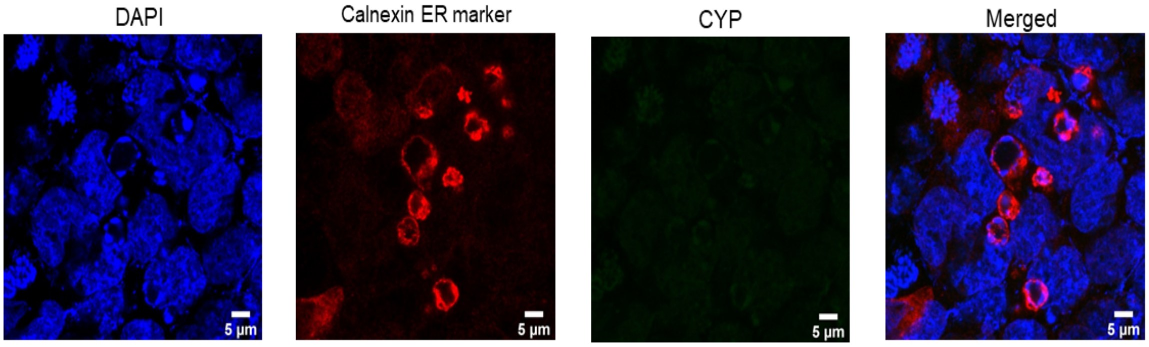

Figure S2. Representative Western blots indicating the impact of cell heme level on the expression levels of CYP3A4 and 2D6 in HEK293T or HEPG2 cells. Representative Western blots showing CYP expression levels in cells treated according to the indicated conditions. Points in the graphs are from three individual cultures with the mean ± S.D. as indicated.

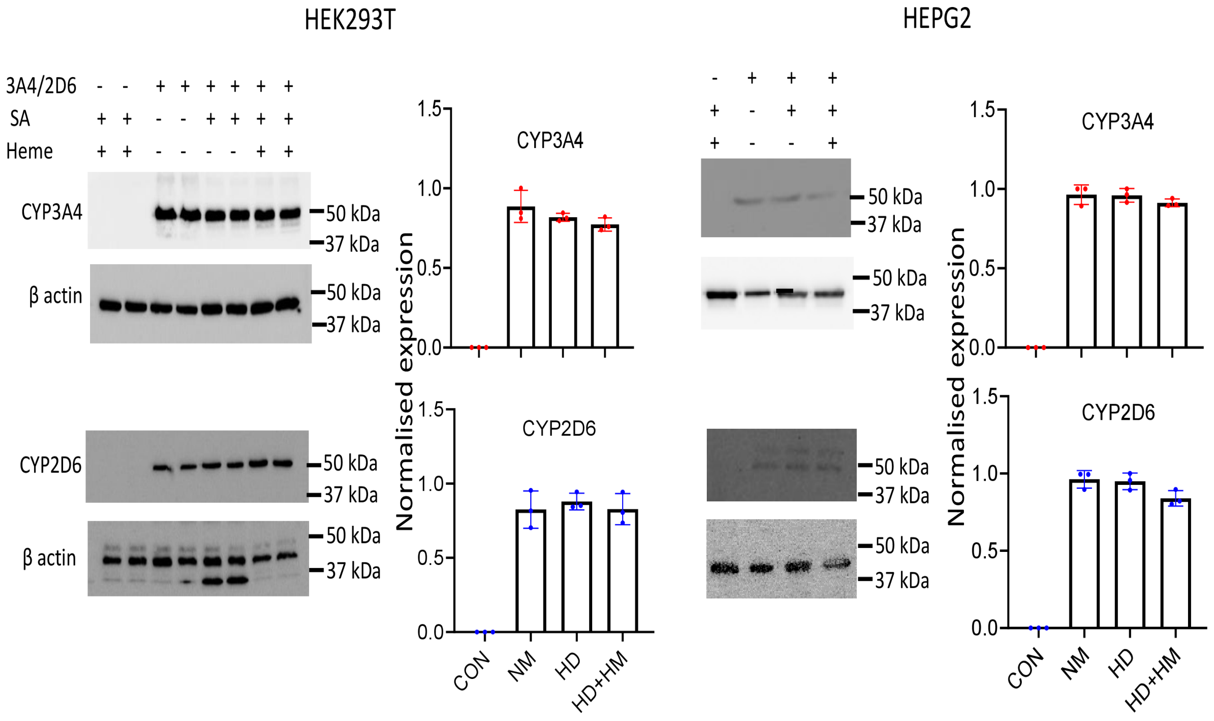

Figure S3. Representative Western blots indicating the impacts of siRNA GAPDH knockdown and the expression of HA-tagged wild type or H53A GAPDH on the levels of total GAPDH and CYP expression. HEK293T cells were transfected to express FLAG- and MYC-tagged CYP3A4 (left) or CYP2D6 (right). The blots shown are representative of 3 independent trials.

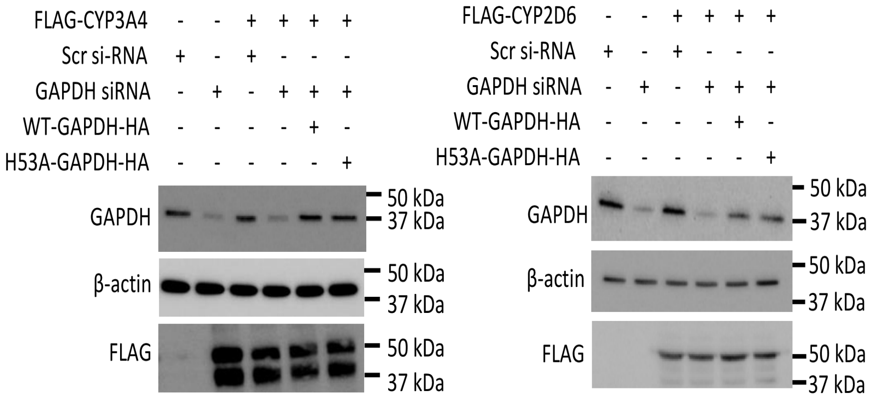

Figure S4. Representative Western blots indicating the impacts of siRNA GAPDH knockdown and the expression of HA-tagged wild type or H53A GAPDH on the levels of total GAPDH and CYP expression. GlyA-CHO cells were transfected to express FLAG- and MYC-tagged CYP3A4 (left) or CYP2D6 (right). The blots shown are representative of 3 independent trials.

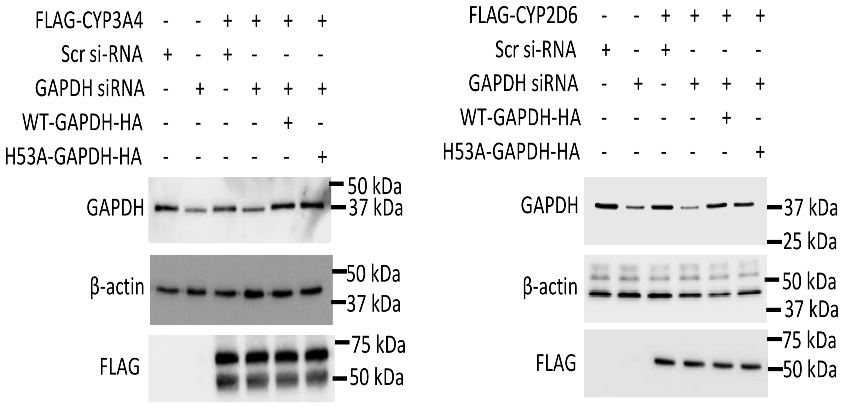

Figure S5. Effect of Hsp90 inhibitors on the expression levels of CYP3A4 and 2D6. Heme-depleted HEK293T or GlyA-CHO cells were transfected to express FLAG and MYC-tagged CYP3A4 or 2D6 and then were given heme precursors without or with the Hsp90 inhibitors radicicol, ganetespib, or AUY922, and then cells were harvested after an additional 6-8 h of culture. Supernatants analyzed here are the same as utilized in Fig. 5. Top panels, representative Western blots. Lower panels, relative levels of CYP expression as determined from the band intensities and normalized to the β-actin band intensities. Values are from three independent experiments.

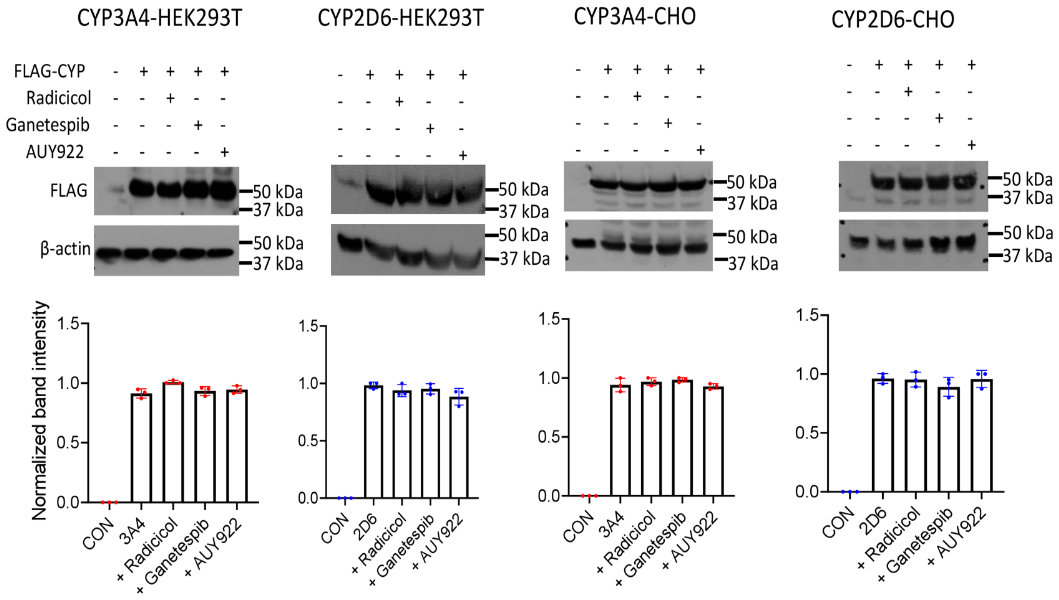

Figure S6. Combined GAPDH knockdown and Hsp90 inhibition does not impact CYP3A4 expression.

Heme-depleted GlyA-CHO cells underwent siRNA GAPDH knockdown or received scrambled siRNA. After 24 h the plates were re-transfected to express FLAG and MYC-tagged CYP3A4. After 30 h the cells were treated with 10 μM Radicicol and then were harvested after a further 6-8 h of culture. In some cases, cells were not transfected to express CYP3A4, and/or were given only scrambled siRNA, siGAPDH, or radicicol treatment alone as indicated. Cell supernatants equal total protein) underwent SDS-PAGE and Western blotting to compare the expression levels of the indicated proteins. Results shown are representative of 3 replicates.

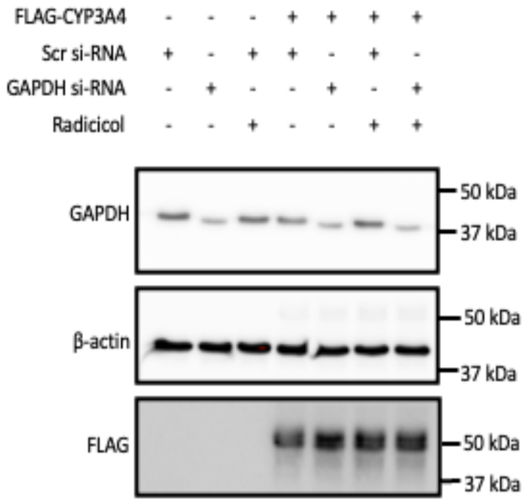

Figure S7. Representative Western blots indicating the relative levels of Hsp90 or GAPDH associated with FLAG and MYC-tagged CYP3A4 or 2D6. HEK293T cells or HEPG2 cells were cultured either in normal media and serum (NM) or with medium containing SA and heme depleted serum (HD) and were transfected to express FLAG and MYC-tagged CYP 3A4 or 2D6. After 36 h cells were harvested and the supernatants (equal protein) underwent FLAG Ab pulldown. In some cultures, hemin was added 3 h before cell harvest (HD + HM).

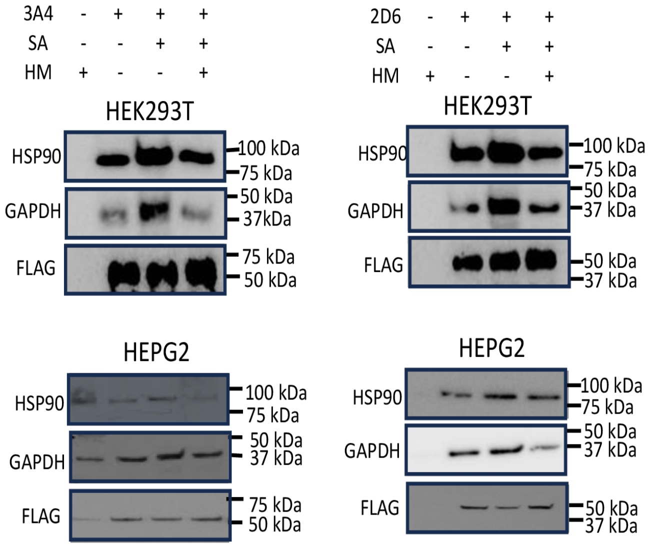

Figure S8. Expression and activity levels of CYP3A4 and 2D6 in mouse liver and their association with GAPDH and Hsp90. Supernatants from the livers of 3 mice were prepared and individually analyzed. CYP activities were measured along with the CYP expression levels by Western blot, and the GAPDH or Hsp90 associations with either CYP were examined by pulldown using anti-CYP3A4 and 2D6 antibodies, with the GAPDH and Hsp90 bands normalized to the CYP band intensities.

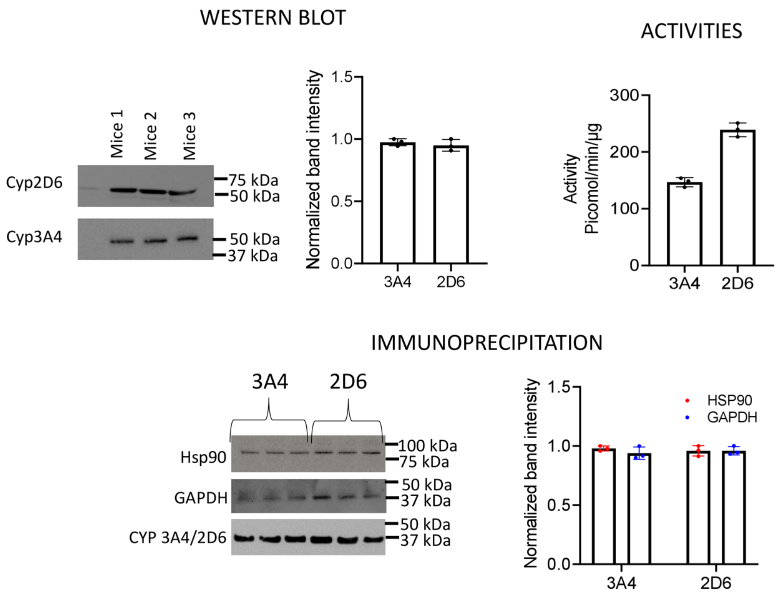

Supplement: Figures S1–S8 [file mmc1.pdf]
